# Application of Service Oriented Architecture for Sensors and Actuators in District Heating Substations

**DOI:** 10.3390/s140815553

**Published:** 2014-08-21

**Authors:** Jonas Gustafsson, Rumen Kyusakov, Henrik Mäkitaavola, Jerker Delsing

**Affiliations:** Division of EISLAB, Luleå University of Technology, 971 87 Luleå, Sweden; E-Mails: rumen.kyusakov@ltu.se (R.K.); henrik.makitaavola@ltu.se (H.M.); Jerker.Delsing@ltu.se (J.D.)

**Keywords:** district heating, wireless, substation, WSN, SOA, architecture

## Abstract

Hardwired sensor installations using proprietary protocols found in today's district heating substations limit the potential usability of the sensors in and around the substations. If sensor resources can be shared and re-used in a variety of applications, the cost of sensors and installation can be reduced, and their functionality and operability can be increased. In this paper, we present a new concept of district heating substation control and monitoring, where a service oriented architecture (SOA) is deployed in a wireless sensor network (WSN), which is integrated with the substation. IP-networking is exclusively used from sensor to server; hence, no middleware is needed for Internet integration. Further, by enabling thousands of sensors with SOA capabilities, a System of Systems approach can be applied. The results of this paper show that it is possible to utilize SOA solutions with heavily resource-constrained embedded devices in contexts where the real-time constrains are limited, such as in a district heating substation.

## 1. Introduction

To apply a System of Systems approach, where sub-systems of different nature will interact, system-wide communication must be enabled. A system-wide communication architecture enables more efficient resource usage where measurements and services can be reused in a “wrap-and-reuse” fashion instead of the expensive “rip-and-replace” method used today. The introduction of a system-wide communication does not come easily and a number of limitations have to be overcome. In this paper, we evaluate the energy and timing cost of utilizing a high level SOA architecture on resource-constrained wireless devices - a research problem with important implications in wireless sensor/actuator network research [1]. The time and energy cost induced by the SOA-on device approach should be weighed against the cost of using a multitude of specialized solutions, requiring advanced middleware that often requires frequent “re-calibration” or updates.

As an example application, district heating suppliers can introduce new customer services and high-level control to improve district heating (DH) system efficiency. As district heating is a widespread heating method in Europe, and growing in, e.g., China, large financial benefits are possible through improved monitoring and control.

Combining sensors, meters and actuators distributed throughout a DH system with heat plant control and enterprise systems will enable completely new possibilities for system wide control and optimization. Customer services such as remote meter reading, fault detection and heat-system control can be significantly improved and provided directly by heat suppliers or third party companies.

Within a district heating system (and other energy distribution networks such as the electricity grid) there are often many independent data collection systems for billing purposes and automatic meter reading (AMR), e.g., power line communication and proprietary radio solutions. These are often non-flexible and expensive methods that requires much support from different maintenance-staff. By the introduction of SOA technology a more generic approach is achievable, where meters can integrate directly with higher level system without the use concentrators or adapter solutions.

The SOA solution will also enable the possibility to integrate the DH meter reading system with other building monitoring & control and home automation, e.g., ventilation, alarms and lightning, creating a System of Systems architecture. A larger scale system of systems view of our approach is in the context of optimization of heat production, where SOA enabled devices at end-customers can be orchestrated in real-time to reduce peak-production at the heat plants.

We now present a short introduction to the key components of our proposed approach.

### 1.1. Service Oriented Architecture

Service oriented architecture (SOA) is seen as a promising technique to bridge the gap between various industrial devices and enterprise applications [2]. Closer integration between ubiquitous embedded systems such as wireless sensor nodes and high-end systems could lead to higher flexibility in process optimization and evolution. The application of this concept has been a research objective of the European ITEA projects Sirena [3], Soda [4] and EU framework project Socrades [2] which had great support from the large industrial actors such as ABB, SAP, Schneider Electric and Siemens. As part of the outcome of these projects, a high-level architecture built on top of the web service technology was proposed. A key element in this architecture was the use of the same communication protocols on all levels of the industrial enterprise - business, manufacturing execution systems (MES) and the shop floor. As the deployment of the web service technology on resource-constrained devices leads to unacceptable overhead in terms of RAM, CPU and network usage the use of intermediate components such as gateways and mediator services was suggested. However, the introduction of additional hardware and software modules to the system increases its complexity and maintenance costs. As opposed to this approach, the solution presented in this paper deploys the web service interface directly on the resource-constrained wireless sensor nodes. To lower the resulting overhead, a newly emerging encoding technique for the XML service messages was employed.

### 1.2. Wireless Sensor Networks

To enable easy installation and avoid “wirenests”, a wireless approach is preferable. In this paper, we are advocating a wireless solution based upon open well-established standards. As there are a number of sensing and actuating devices to be interconnected, a Wireless Sensor and Actuator Network (WSAN) approach is appropriate.

A WSAN consists of a number of wireless sensor platforms (nodes) distributed over an area to monitor surrounding conditions, e.g., temperature or flow, or to control a physical property such as pump speed. The sensor platforms can communicate wirelessly, generally using radio technology. The number of connected nodes (sensor platforms) can vary from just a few up to several thousands [5].

During the last few years, the technology of 6LoWPAN [6,7] has enabled the next generation Internet protocol IPv6 to be used on top of IEEE 802.15.4 low-power radio modules. This has enabled the use of standard Internet-suite communication protocols (TCP/IP family) and cheap, resource-constrained devices [8]. With the ability to use TCP/IP at the sensor level, the need for gateways with advanced middleware solution is obviated. It also enables the use of standardized SOA-technologies directly on the sensor platforms. However, most existing SOA-suites were developed to function on powerful desktop or server computers connected with high-bandwidth networks [9], which is a major challenge when deploying SOA on lightweight embedded devices.

In this paper, the focus is on sensor level performance, and not network performance. Network performance and simulation of complete network are very interesting and important aspects, but we did not find them suitable to include in this paper, and hence left it out for future work. In addition, security aspects are an important factor when it comes to large scale deployment, this has also been left out of paper, since we consider it to be a too complex question to fit inside the boundaries of this paper.

### 1.3. District Heating

District heating is a technology in which a central heat plant (boiler) heats a distribution medium (usually water) to be distributed in an often city-wide underground pipe system (distribution network). This system can achieve a higher fuel efficiency than the use of independent boilers in each house, and it also reduces installation cost and complexity in connected properties because no local boiler has to be installed [10].

The distribution medium temperature (T_ps_) is controlled by the heat supplier; it is normally increased in response to decreased outdoor temperatures to compensate for the increased heat demand and lowered at lower heat loads to limit heat overproduction and thermal losses.

**Figure 1.**
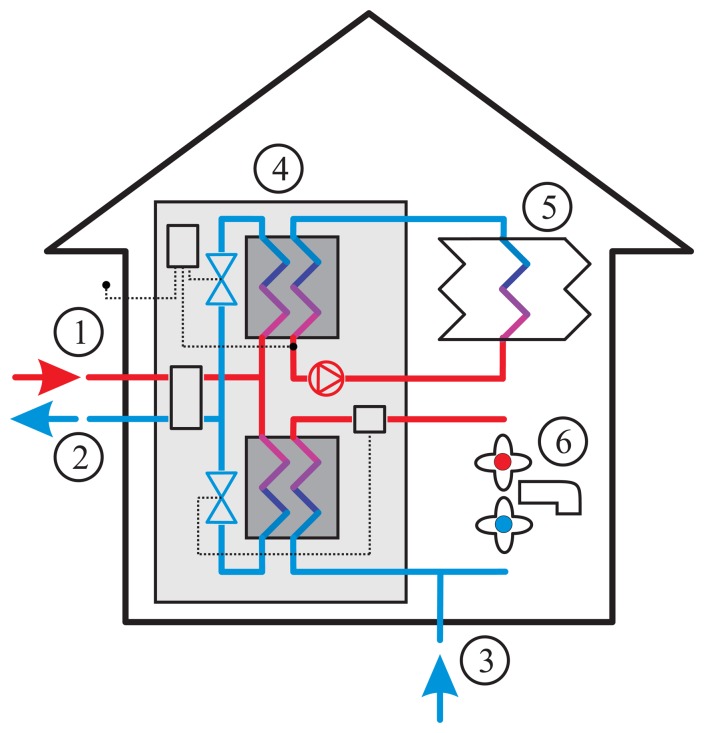
1, Incoming district heating. 2, Returning district heating. 3, Incoming cold tap water. 4, District heating substation. 5, Radiator system. 6, Water tap.

In DH-connected buildings, the heat carried by the distribution medium is transferred to house internal housing systems. In most modern DH-systems the distribution medium is separated from the internal heating systems (space heating and tap water heating) in a so-called district heating substation (DHS). The heat is transferred through a setup of heat exchangers to heat both the tap-water and space heating systems. A simplified DHS installation can be seen in Figure 1. In the substation, there are also a number of sensor and electro-mechanical actuators that control the heat transfer rate to maintain a stable indoor temperature. For billing purposes, a heat meter is also found in the substation to measure how much energy the customer uses.

These devices have traditionally been statically hardwired in a predefined setup using proprietary protocols (analog or digital). In the last few years, wireless indoor and outdoor temperature sensors have become more common; however, they are still using proprietary communication methods. The other devices are still hardwired to a control unit that interconnects the peripheral devices and hosts the control algorithms.

In [11], a WSN-enabled DHS control system was created using the 6LoWPAN/IPv6 technologies, where the control calculations were performed on the wireless sensor platforms. In this paper, we are evaluating the possibility of adding a service oriented architecture to the system presented in [11] in order to improve its flexibility and interoperability.

### 1.4. Paper Structure

The rest of the paper is structured as follows: Section 2 provides overview of the related work and how the current approach relates to the state-of-the-art research in the area. In Section 3, we first explain the field of application, which is district heating and what challenges and opportunities we see. Further we go through the technical specification of the wireless sensor network platform and network architecture used in the experiments. Section 4 provides detailed description of the protocol suite and service oriented architecture used in the setup. In Section 5 we give an overview of the data standards that are of interest for the sensor data acquisition field and present an example of how we structure the data. In Section 6 an overview of the experimental setup hardware is presented. In Section 7 the experimental results are presented and discussed. Section 8 concludes the paper and analyses the applicability of the approach.

## 2. Related Work

The idea of using Service Oriented Architecture for systems level integration and systems evolution has already been investigated and proved beneficial for improving the resource utilization and extensibility. A study on application of SOA for enabling evolvable production systems has been presented by Candido *et al.* [12]. This work proposed a high level architecture for customizable production infrastructure where system functionality is discovered and composed dynamically based on existing services and semantic meta-data in the form of ontologies. A similar approach is presented for the domain of maritime surveillance by Parlanti *et al.* in [13]. They introduced a SOA-based platform that is targeted at the integration and interoperability of heterogeneous systems owned by different stakeholders. In this article we applied similar approach for the domain of district heating and extended the aforementioned architectures with concrete measurements and application of device level web services.

A work that is more closely related to the discussions we present here is given in [14]. The authors of this work were proposing an approach for integration of wireless sensor nodes in a service oriented facility management architecture. They suggested the use of SOA for integration of various systems such as heating, ventilation and air conditioning (HVAC), security, electricity systems *etc.* As opposed to our approach, they were using gateway devices for accessing the WSNs and the sensor nodes were not equipped with web service interfaces. Moreover, their work did not provide validation of their architecture through simulations or prototype experiments. Another difference is that, while their architecture inherited many principles from the Sensor Web Enablement (SWE) framework [15], their approach was not compliant with the SWE specifications.

The Sensor Web Enablement (SWE) framework is a major building block in the Service Oriented Sensor Web architecture proposed in [16]. However, the authors of this architecture implement and evaluate the SWE specification on a gateway and the communication with the sensor devices is still based on ad-hoc protocols.

## 3. Functionality and Architecture of Future DH Substations

In this paper, we have chosen to use district heating as an example application area because we know that in the DH-industry there is a need for open, system-wide solutions that can evolve over time.

In the competition for heating customers, it is of the utmost importance to maintain a high quality of service (QoS) at a competitive price. Historically, district heating has been known to be a reliable and convenient heating method that requires very little or no maintenance at the customer level. However, the ability of both customers and heat suppliers to obtain useful information on how and where the energy is used within the building has been limited (which is not a unique problem for district heated houses). The space heating control system(s) has also been difficult to calibrate for maximum performance. Systems for high-level monitoring and control that includes DHS functionality have been non-existent until recently. By introducing wireless sensors, which are available for a multitude of purposes, the functionality and performance of the substation (and indirectly the complete DH system) can be improved. The use of loosely coupled sensors and actuators with the possibility to reconfigure and interconnect them can enable new types of services.

Over the last 5–10 years, several new inventions have emerged that would increase the functionality and QoS for both heat suppliers and customers; see [17–20] for a selection of promising recent research results. Each of these results can be implemented today using specialized hardware installations, making it expensive for the customer to utilize them all. For that reason we suggest the utilization of open, vendor-independent standards for inter-systems communication. Specialized solutions using proprietary protocols and hardware are by default incompatible with products from other manufacturers. This often leads to an expensive vendor lock-in for both energy companies and property owners.

Using the well-established open communication suite of TCP/IP at the sensor level enables a reliable communication architecture that will support high-level SOA integration. However, as previously discussed, most SOA technologies today are too complex for low-power, highly resource-constrained embedded devices. Using emerging encoding technologies like EXI (see Section 4 for further details), it becomes possible to apply open and SOA compatible solutions applicable for WSN installations.

### 3.1. Wireless Sensor Network Architecture

The sensor platform used in the underlying experiments to this paper is the so-called Mulle v6.2 platform, modified to support 2.4 GHz radio frequency. The Mulle platform originated from the EISLAB division at Luleå University of Technology, but it is now commercially available through Eistec AB [21]. The Mulle is designed with low-power consumption in mind, but it is still powerful enough to support, e.g., a TCP/IP stack. Both TinyOS [22] and Contiki [23] lightweight operating systems are ported to the Mulle; in this paper, we have used TinyOS exclusively.

**Figure 2.**
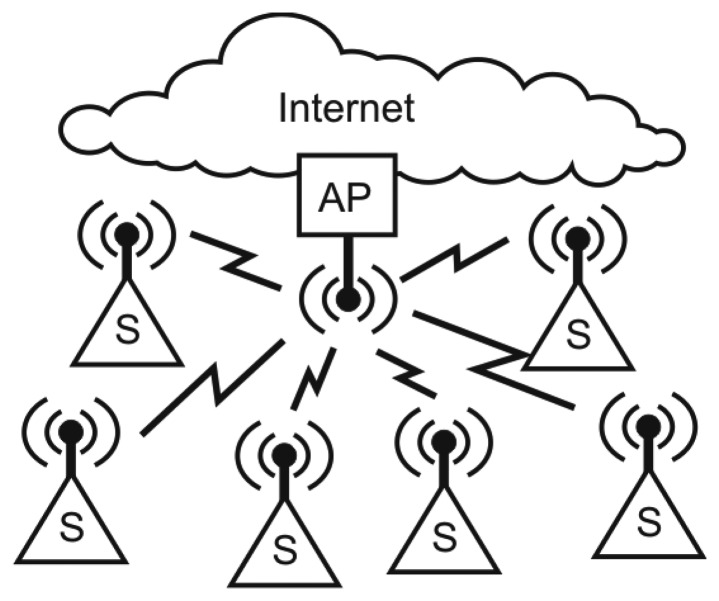
Sensor network. Sensors and actuators are connected to wireless sensor platforms (S). The sensor platforms can advertise their services within the sensor network and on-line as they utilize the Internet Protocol (IPv6). All on-line traffic is routed through the access point (AP).

The wireless sensor platforms connect to the Internet through an Internet access point (AP) as depicted in Figure 2. The AP also acts as an edge-router for the 6LoWPAN network and supports the following interfaces: Ethernet, 3G/GPRS, IEEE 802.15.4 and Bluetooth communication. The operating system running the Access Point is μCLinux [24], running on a Blackfin [25] micro-controller from Analog Devices.

### 3.2. Network Technologies

**Figure 3.**
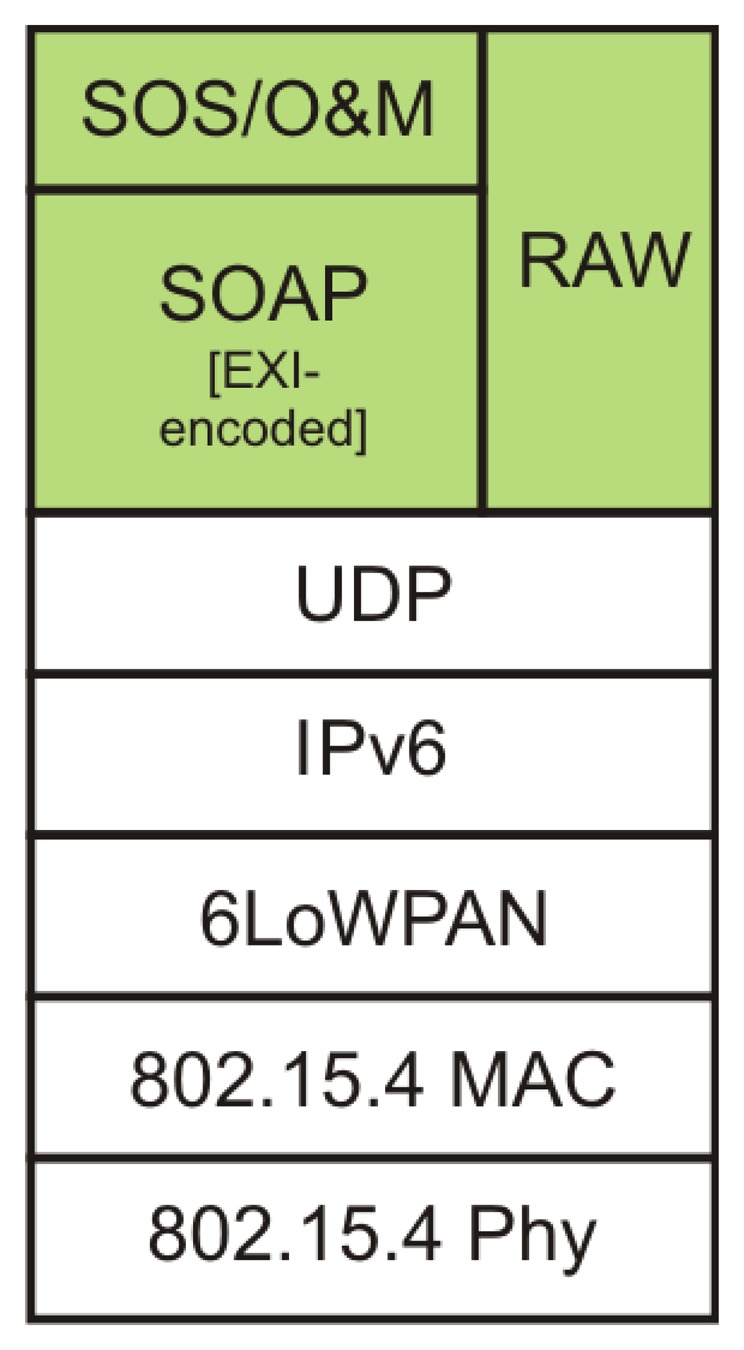
The complete network protocol stack used in the setup.

The network connection between the sensor platform and the access point relies on the PHY and MAC layer specified in IEEE 802.15.4 [26]; see also Figure 3. The standard supports multiple radio frequency band profiles, in the experiments conducted for this article we used the 2.4 GHz profile with a bet rate set to 250 kb/s.

On the network layer, we used the next generation Internet Protocol (IPv6) [27], which is already supported by most routers on the Internet today. This makes the WSAN in the DH substation globally accessible for systems connected to the Internet.

The IPv6 header has fixed length of 40 bytes (320 bits) with a MTU (Maximum Transfer Unit) of 1280 bytes. However, the frame size of IEEE 802.15.4 is only 127 bytes, with 76–116 bytes of payload available, depending on the addressing and security options [7]. This requires fragmentation of the IPv6 packets that is handled by the 6LoWPAN adaptation layer [6], found in between the Network- and Link-Layers in the protocol stack, see Figure 3. The 6LoWPAN also handles the header compression of the IPv6 packages to make the transmission over the IEEE 802.15.4 link faster and more resource efficient.

The transport protocol of choice in the experimental setup is UDP. Due to the limited bandwidth and computational power of the resource-constrained sensor platforms, TCP can be experienced as slow, with long round-trip time (RTT) [28].

At the application level, we are using the simple object access protocol (SOAP) to encapsulate messages to be sent and received over the network as further explained in Section 4.

A possible alternative or complementary approach to SOAP would be to use CoAP (Constrained Application Protocol) [29], which is specifically developed to work well with resource constrained systems. However, as parts of the underlying work of this paper were conducted before the CoAP standard was established, we choose not to introduce CoAP any further in this particular paper, but consider it for future work. An overview of CoAP features and comparison with SOAP for embedded systems is presented in [30].

## 4. Web Services

Although there are a number of different open and proprietary solutions conforming to the SOA paradigm, web service technology is the most widely adopted by the industry for at least two reasons. First, it is not encumbered by any patents and licenses but is built upon the same standards and protocols already used and proven in the world wide web. Second, not only are the web service specifications and standards free to the public, but there are also many open source software tools and development kits for building SOA applications in different programming languages. A service message carrier protocol called Simple Object Access Protocol (SOAP) is often used as a means for defining the formatting of service requests and responses and specifying the interchange patterns. Each SOAP message is serialized as an XML document with a predefined structure and can be sent using different network protocols. Although SOAP is independent of the transport protocol used, in practice, the SOAP documents are embedded in the HTTP body or sent directly as a UDP payload. Recent efforts aimed at applying the SOA approach to embedded systems have resulted in the Devices Profile for Web Services (DPWS) [31] specification, where the binding of SOAP to UDP was formally defined. The SOAP-over-UDP [32] supports unicast one-way, multicast one-way, unicast request, unicast response, multicast request and unicast response message patterns through the use of WS-Addressing specifications. The web service implementation used in our experimental setup was based on SOAP-over-UDP on top of an IPv6 network layer, and the tests included the unicast request and unicast response message exchange patterns.

Moritz *et al.* investigated the use of SOAP on top of CoAP by defining formal binding for the two protocols [33]. They showed that the new binding provides certain benefits such as lower delays and overhead compared to HTTP binding and higher reliabilities than simply using UDP. However, the SOAP-over-CoAP binding [34] has not progressed further and was not adopted as a standard.

The use of web services for inter-system communications fosters interoperability among heterogeneous and complex business and industrial processes. The benefits achieved result from the service's vendor independence and ability to interconnect different operating systems, programming languages and hardware platforms. However, the main building block in the web services specifications, namely, Extensible Markup Language (XML), is too verbose and resource consuming to be used for battery powered resource-constrained embedded devices. The size of the XML documents used to carry the service messages is the main limiting factor preventing the application of the SOA approach in the embedded domain, especially over wireless links. Nevertheless, recent advancements in binary encodings for XML have achieved performance that is very close to that of highly optimized data formats. The use of these binary encodings enables XML processing for severely constrained wireless sensor nodes. Evaluations of several high-performance XML encoding formats have been made available by XML Binary Characterization Working Group (Efficient XML Interchange Measurements—http://www.w3.org/TR/exi-measurements/). In addition, surveys comparing different binary representations for use in web service messages are investigated in [9,35]. The highest efficiency and processing speed is achieved using the Efficient XML Interchange (EXI) format [36] developed by the W3C Binary XML Working Group. It is based on information theory and formal languages and uses knowledge of the structure of the XML documents to achieve compact representations for both document meta-data and payload. Each EXI document consists of a header and a body. The header defines different encoding/decoding parameters that affect the compactness, processing efficiency and memory usage when processing the document. An important parameter is the use of an XML schema that sets constraints on the structure and content of the document. The schema information must be available before the EXI encoding/decoding, and it should be either statically set or communicated in advance using schema languages such as W3C XML Schema. Schema-enabled EXI processing is much faster and results in smaller EXI documents compared to schema-less processing. Table 1 shows the sizes of the request and response messages used in our experiment encoded using plain XML, schema-less EXI and schema-enabled EXI.

In our experimental setup, we used the EXIP open source implementation (Efficient XML Interchange Processor—http://exip.sourceforge.net/) to parse and serialize EXI messages, as it was specially designed for resource-constrained embedded systems [37]. Our prototype implements schema-less processing but schema-driven encoding and decoding are also possible.

Due to the pre-conditions while doing the experiments for this paper, we did not have the opportunity to run schema-enabled EXI transmissions. However, a compression rate exceeding 90% compared to uncompressed XML when using strict mode schema-informed EXI serialization is reported by Altmann *et al.* [38], these results are comparable to what we would expect in our set-up.

## 5. Data Models

We find it important to follow available standards as much as possible. This also applies to the semantics of the XML structure describing the service messages. The Open Geospatial Consortium (OGC) is working on standards for enabling sensor communication over the web. The Sensor Web Enablement (SWE) [15] framework includes a number of standards under development to support developers making sensors and actuators discoverable, accessible and usable via the web. We identified Observation and Measurement (O&M) [39], Sensor Observation Service (SOS) [40] and SensorML [41] as the most essential for our application domain.

- **O&M** specifies an abstract data model to be used for physical observations and measurements.
- **SensorML** defines standard syntactical constructs to specify the functionality and available services on a particular sensor platform.
- **SOS** provides an interface for managing sensor platforms and retrieving sensor data.

The service messages included in our proof of concept experiment followed the formatting rules defined in O&M and SOS standards. However, it does not include the messages defined by SensorML. A complete description of the sensor nodes, including detectors, physical structure, input/outputs could also be added to the system using SensorML as a future work.

**Figure 4.**
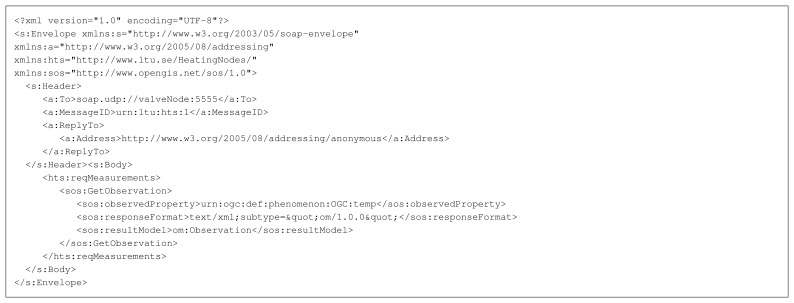
SOAP service request message in XML text format. EXI-encoded total size: 451 bytes.

**Figure 5.**
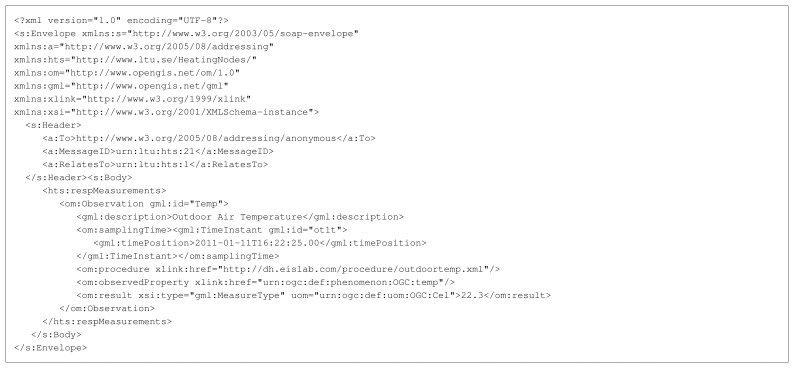
SOAP service response message in XML text format. EXI-encoded total size: 651 bytes.

Figure 4 shows the complete request message used in the experimental setup. The responding platform parses the message and encodes a response message using the O&M standard, which can be seen in Figure 5.

## 6. Experimental Setup

To test the hypothesis of using a SOA in a low-power sensor network, a simple experimental setup was created. Two sensor platforms were programmed to be used in the experiment. One platform was programmed to request temperature information from the other platform using standard Web services. We refer to the requesting platform as the *Requester* and the responding platform as the *Responder*.

The Web service interface of the sensor platforms is a simplified SOAP engine that uses the Berkeley Low-power IP Stack (BLIP) of TinyOS to sent and receive UDP packets with SOAP messages as a payload (see Figures 4 and 5 for example messages). Instead of using plain XML representation the SOAP messages were encoded in EXI to reduce their size. The EXI encoded UDP payload is parsed by the EXIP library to extract the SOAP fields. The values of these fields are then passed to a protocol state machine that reacts on the content of the SOAP message - store the value, send a response, send an error when problems *etc.*

**Figure 6.**
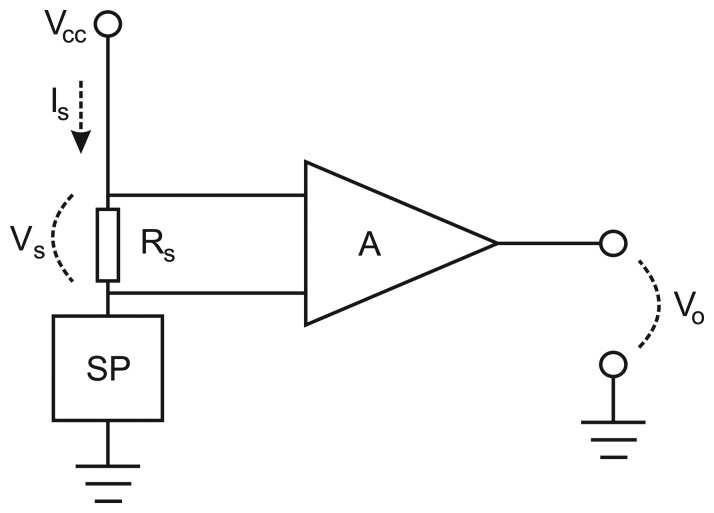
Current measurement setup. SP indicates Sensor Platform and A indicates amplifier. V_o_ is measured at 50 kHz by an A/D data acquisition card.

The current usage of the sensor platforms was measured using the simple measurement setup depicted in Figure 6. A small shunt-resistance (R_s_) of 1Ω was connected in series with the sensor platform, and the voltage (V_s_) generated across R_s_ was amplified 100 times and fed into a data acquisition card. The data acquisition frequency was set to 50 kHz to record all sensor-platform activity. From the recorded data, the current usage can easily be calculated using Ohm's law in combination with the amplification factor A; see Equation (1).

**Figure 7.**
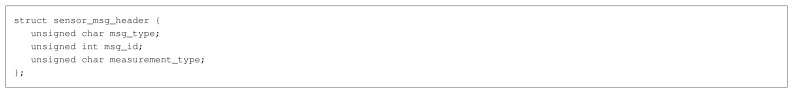
RAW request message. Transmission size: 4 bytes.

**Figure 8.**
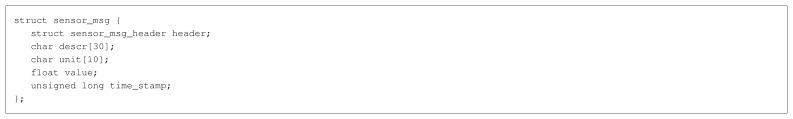
RAW response message. Transmission size: 52 bytes.

The OGC-compliant Sensor Web Enablement (SWE) service requests and responses were compared against a minimalistic custom data transfer (RAW). In our setup RAW represents the minimal bytes sequence needed for carrying a set of structured data. The RAW message corresponds to a predefined C structure and is loaded without tokenizing the bit stream. The RAW format we chose has severe limitations that makes it impractical for real-world usage but we find it as an useful base for comparison as it represents the minimum possible size of a message. Figures 7 and 8 show the RAW-message constructs. In order to be usable in practice, the RAW format must be extended to correctly handle differences in endianness between hosts. Also adding the possibility to transmit different data types and varying number of fields and support for meta-data fields is needed for application beyond a specific use case.

(1) $$I_{s}=\frac{V_{o}}{A\cdot R_{s}}$$

To get an idea of time and energy required for serializing/sending and receiving/parsing EXI-encoded XML-messages the current usage was measured throughout the operation. Because the RAW transmissions do not require serialization like the EXI-encoding, the total transmission time will be significantly reduced for RAW-message operation. The smaller size of the RAW messages (maximum of 52 bytes) also obviates the need for message fragmentation by the 6LoWPAN layer. Thus, only one IEEE 802.15.4 packet is needed to transmit the required RAW data. In Section 7, the measurement results are analyzed further.

## 7. Results

The measurement results were divided into 4 independent processes to identify how much they contribute to the total time and energy usage for one interaction:

- **Serialization, SR.** Encoding and serializing the message using the EXI-standard.
- **Sending, SE.** Sending the EXI-encoded message using IPv6/6LoWPAN.
- **Receiving, RE.** Receiving the message.
- **Parsing, PA.** Parsing the received EXI-encoded message.

Depending on whether the current-measurement is performed on the Requester or the Responder, the order of the processes mentioned above will vary.

**Figure 9.**
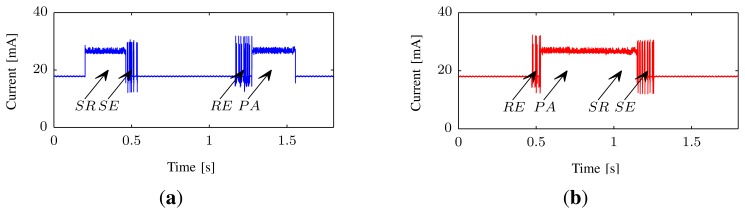
Current usage over time, at requester and responder, during an EXI-transaction. SR—Serialize, SE—Send. RE—Receive, PA—Parse. The sensor platform is not put into sleep mode in between activities; thus, current usage is ∼18 mA while idle. **(a)** Requester; **(b)** Responder.

For the Requester, we also measure the total time from the initiation of serialization to the parsing of the received result also see *t_Return_* in Figure 9a; we call this the *Return*-time. At the Responder, we measure the time from the reception starts until the transmission of the response message finishes, which we call the *Response*-time; see also *t_Response_* in Figure 9b.

**Figure 10.**
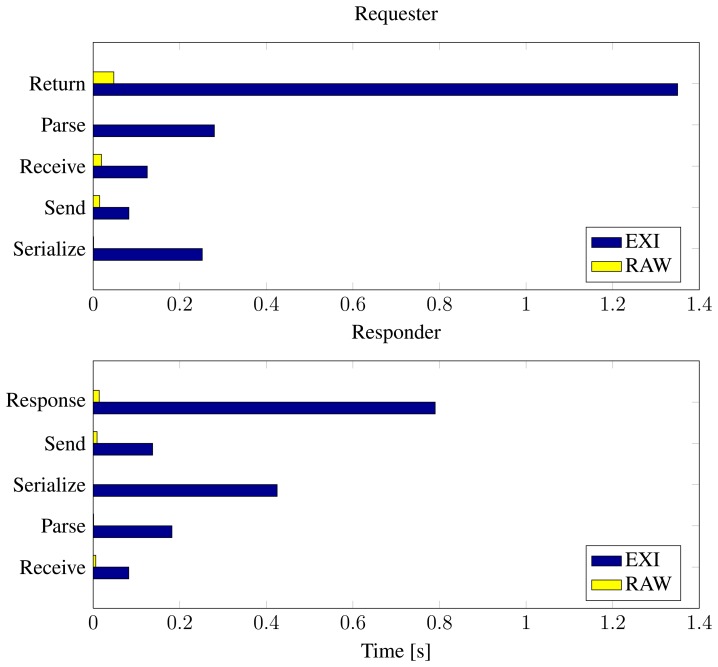
Visual presentation of Table 2, average processing times in communication process.

In Table 2, the timing results for serializing, sending, receiving and parsing messages at both the Requester and the Receiver are presented, these results are also visualized in Figure 10. These measurement results reveal that it is the parsing and serialization of the EXI-messages that requires the most time and energy in the communication process.

Compared to sending a minimal RAW payload over IPv6/6LoWPAN, the EXI-encoded SOA solution requires more energy and time to complete the operation of requesting the temperature. The RAW method return time is less than 0.5% of the EXI-encoded return time. However, the longer return time of the EXI solution should be weighted against the cost of having several individual specialized systems requiring specialized maintenance and support.

### 7.1. Time and Energy Constrains

With a return time of ∼1.3 s, the SOA approach used in this paper might not be suitable for use in real-time processes with significant time constraints. However, in our target application (DH-substations), a return time of 1.3 s does not have any direct implications for measurement and space heating control because the real-time requirements are low. For hot-water control in buildings without hot water circulation (HWC), a shorter return time might be advantageous to avoid temperature oscillation in tap water. Today, hot-water control in smaller properties without HWC is usually controlled by self-acting mechanical solutions; as this is not within the primary application area, we consider it to be a marginal issue.

Using SOA on battery-powered sensor platforms will affect the life expectancy of the sensors by increasing the computational and transmission time. To evaluate the effect of increased current consumption, an extrapolating calculation was performed.

The outdoor and indoor temperature sensors connected to a DH substation are typical devices where a battery is the only reasonable power source. Because these temperatures do not change rapidly, a sampling frequency of e.g., four measurements per hour is mostly enough for measurement and control purposes. Because the control system in a district heating substation is working continuously, there is no need to ask specifically for a certain temperature each time it is required. A subscription method where the sensors involved automatically send their temperature readings to subscribing receivers is instead considered. Sending the temperature using an EXI-encoded O&M message to one subscribing receiver four times per hour requires the WSN platform to wake up from deep sleep (idle mode), read the temperature, serialize and send the message.

The energy *Q_s_* used by a sensor platform for a specific time *t* can be calculated by Equation (2). The serialization of the temperature measurement takes ∼425 ms at a reasonably constant current consumption, *I_s_* of ∼27 mA (see Figure 9) at a V_cc_ of 4.2 V. This results in an energy usage of ∼48 mWs for one response message.

**Figure 11.**
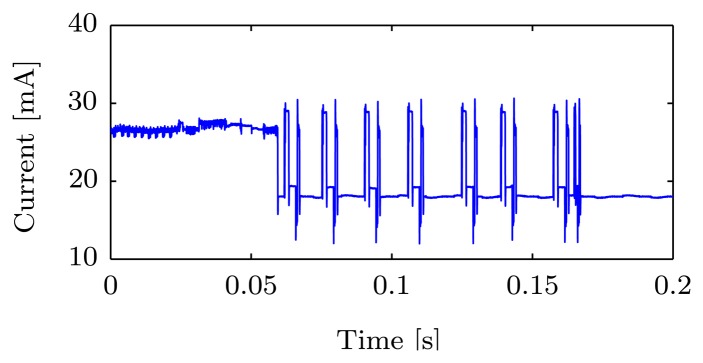
The figure shows the current consumed during transmission of an IPv6 packet. The 6LoWPAN fragmentation of the IPv6 packet is clearly visible. The IPv6 packet is divided into 8 6LoWPAN frames.

Figure 11 shows the current usage of a transmission in detail; observe the 6LoWPAN fragmentation (eight spikes) of the IPv6 package. The energy usage for the transmission is also calculated using Equation (2). Sending the serialized payload of 651 bytes will use approximately use 10 mWs. Thus, one complete temperature serialization and transmission uses approximately 48 + 10 = 58 mWs. Sending four messages per hour results in 238 mWs per hour which equals ∼65 μW in average power usage needed for serialization and transmission. In addition, 12 μW are consumed while the sensor platform is in deep sleep between transmissions, and there is also a necessary hardware-dependent energy for sensor reading, which we estimate to 8 μW on average in this example. The total average power consumption *P* can thus be approximated as 85 μW (65 + 12 + 8).

(2) $$Q_{s}=V_{\text{cc}}\int_{0}^{t}I_{s}(t)\text{dt}$$

We can now estimate how much energy is needed to keep a this sensor platform alive for a specific time. Because heat meters in district heating substations need to be re-calibrated at 5 or 10 year intervals (Due to country specific rules and regulations), it would be convenient to have the battery powered devices running for at least the same amount of time between battery changes. The energy needed to keep a sensor platform alive for 5 years (43800 h) will require at least ∼3.7 Wh; see Equation (3). However in practice, we realize that a somewhat bigger battery is required since there will be energy losses within the battery. Approximately twice the theoretical capacity should in practice be enough. Commonly found batteries in commercial heat meters are often around 5 Ah at 3.6 V ( 17 Wh) with a physical dimension of around 6 × 3 cm. Adding roughly 2 Ah to this battery, either by adding another battery or replacing it with a bigger one would in most cases not require any change to the physical dimensions of the heat meter.

(3) $$Q_{\text{tot}}=t_{\text{life}}\cdot P=t_{\text{life}}\cdot \left(P_{\text{tx}}+P_{\text{stop}}+P_{\text{temp}\cdot \text{read}}\right)$$

### 7.2 Memory Usage

In addition to the time and energy constrains, wireless sensor nodes have limited amounts of programming and random access memory (RAM). For example, the Mulle node used in our experimental setup features 512 kB of ROM and 47 kB RAM. The EXIP library and the service engine handling the SOAP messages occupy almost 80 kB of programming memory. The RAM used is allocated during the EXI message processing and is freed immediately afterwards to be made available for the IP stack routines. Although the RAM consumed by our web service solution can be safely shared and reused by other modules running on the sensor node, its size is relatively large, between 7.1 and 9.1 kB for serialization and between 7.7 and 9.8 kB for parsing of the EXI messages.

The measured ROM and RAM consumption can be seen as an upper limit for this particular case, as we have not implemented any optimizations on the code, and we have not used any optimization parameters for the compiler. In a simple test to support this statement, we changed the dimension of several 32 bit integer variables in the EXIP library to 16 bit integers, assuring that the correctness of the execution would be preserved. This change alone resulted in between 0.5 kB and 0.8 kB less dynamic memory used during service request/response execution.

### 7.3. Comparison with Related Work

It is desirable to compare the timing, energy and memory measurements presented in this section with similar approaches in the literature in order to provide a sensible context for evaluation. Unfortunately, there are not many studies using standard Web services on sensor nodes and providing concrete measurements of the system properties. One such study [42] reports response time overhead for Web services of about 2.5 times, *i.e.*, the SOAP-based Web service interactions take about 2.5 times longer to complete compare to conventional plain text representation for a Sun SPOT sensor platform. This measure is much lower compared to our experimental results mainly due to the added overhead for parsing and serialization of the EXI encoding which is not used in the aforementioned study. Furthermore, the time difference between conventional representation and Web services messages becomes larger when only a single sensor reading is included in the message as it is the case in our test setup. This is also shown in the evaluation section of the study on using DPWS in sensor networks [43] where and overhead of about 4 times is reported with a single sensor reading using text encoded SOAP messages.

This comparison highlights the EXI processing as a main source of latency in the proposed architecture. While there are many optimizations that are possible in the EXI processing part of our approach, the application domain of district heating is relatively slow process that does not require real-time communication with the sensors and the currently presented response times are sufficient for its operation.

## 8. Discussion and Conclusion

In this paper, we have evaluated the possibilities of using a service oriented architecture on top of wireless sensor networks in district Heating substations. Our long-term goal in this research is to integrate sensor data from embedded devices in district heating substations with enterprise level systems through the use of service oriented architectures. This will improve the potential sensor data utilization and system flexibility.

The results in this paper demonstrate that it is possible to use SOA on small embedded devices, such as wireless sensor platforms. With schema-enabled EXI parser/serializer in place, the size of transmitted packages will become even smaller, enabling even more constrained and cheaper sensor platforms to comply with the required hardware specifications. With a more optimized software for parsing and serializing EXI-messages, we expect that the return-time can be reduced with at least 50%. When schema-enabled EXI is operational, further reduction in parsing/serializing will be even more efficient, and send/receive times will also be shortened because the message size will be reduced. Still, the processing and transmission time will be longer, and hence the energy requirement will be larger than those in highly specialized solutions, such as the RAW approach used in this paper. However, it is important to put the increased time and energy usage in relation to the cost of keeping several specialized solutions operational and compatible.

The approach of loosely coupled sensors, with the ability to interconnect in a non-predefined way, is controversial in the district heating industry, which is heavily based on strong tradition. However, to offer new services to the customers without expensive additional hardware solutions, it is essential that open and vendor independent solutions are implemented. This was the main approach presented in this article towards a SOA based System of Systems architecture.

To make the presented work available for commercial use, security and data validation issues must be considered. The introduction of security models will affect the expected life-length of battery power sensors as energy is needed to encrypt/validate the data, however, we considered the security issue to be an issue for future work.

The application of the web service enabled wireless sensor nodes presented in this paper is not in any way limited to the area of district heating. Other domains that would benefit from this technology are process automation, facilities management and maritime surveillance to name a few.

## Figures and Tables

**Table 1.** Size of the request and response messages for plain XML, EXI schema-less and EXI schema-enabled encoding.

	Message Size [bytes]	
	
Encoding Type	Request	Response
**XML** (text)	761	1100
**EXI** (schema-less)	451	651
**EXI** (schema)	169	258

**Table 2.** Average processing times in communication process. All results are in milliseconds.

	Requester [ms]					Responder [ms]				
		
Encoding	Serialize	Send	Receive	Parse	Return	Receive	Parse	Serialize	Send	Response
**EXI** (schema-less)	252	82	130	280	1350	82	182	424	130	818
**RAW**	2 *	5	9	*	48	5	*	2 *	9	16

* No serialization or parsing is required in RAW mode; however, the creation of the message structure will require a short period of time.
